# Mucin-1 and its relation to grade, stage and survival in ovarian carcinoma patients

**DOI:** 10.1186/1471-2407-12-600

**Published:** 2012-12-15

**Authors:** Verena Engelstaedter, Sabine Heublein, Anamur Lan Schumacher, Miriam Lenhard, Helen Engelstaedter, Ulrich Andergassen, Margit Guenthner-Biller, Christina Kuhn, Brigitte Rack, Markus Kupka, Doris Mayr, Udo Jeschke

**Affiliations:** 1 Department of Obstetrics and Gynaecology, University of Cologne, Kerpener Straße 34, Cologne, 50931, Germany; 2 Department of Obstetrics and Gynaecology, Ludwig-Maximilians-University, Campus Innenstadt, Maistrasse 11, Munich, 80337, Germany; 3 Department of Obstetrics and Gynaecology, Ludwig-Maximilians-University, Großhadern, Marchioninistrasse 15, Munich, 81377, Germany; 4 Department of Anaesthesiology, Albert-Ludwigs University, Hugstetter Straße 55, Freiburg, 79106, Germany; 5 Institute of Pathology, Ludwig-Maximilians-University, Thalkirchner Str. 36, Munich, 80337, Germany

**Keywords:** Ovarian carcinoma, Mucin-1, CA 15–3 Antigen, CA 27.29 Antigen, Survival

## Abstract

**Background:**

Mucin-1 is known to be over-expressed by various human carcinomas and is shed into the circulation where it can be detected in patient’s serum by specific anti-Mucin-1 antibodies, such as the tumour marker assays CA 15–3 and CA 27.29. The prognostic value of Mucin-1 expression in ovarian carcinoma remains uncertain. One aim of this study was to compare the concentrations of Mucin-1 in a cohort of patients with either benign or malignant ovarian tumours detected by CA 15–3 and CA 27.29. Another aim of this study was to evaluate Mucin-1 expression by immunohistochemistry in a different cohort of ovarian carcinoma patients with respect to grade, stage and survival.

**Methods:**

Patients diagnosed with and treated for ovarian tumours were included in the study. Patient characteristics, histology including histological subtype, tumour stage, grading and follow-up data were available from patient records. Serum Mucin-1 concentrations were measured with ELISA technology detecting CA 15–3 and CA 27.29, Mucin-1 tissue expression was determined by immunohistochemistry using the VU4H5 and VU3C6 anti-Mucin-1 antibodies. Statistical analysis was performed by using SPSS 18.0.

**Results:**

Serum samples of 118 patients with ovarian tumours were obtained to determine levels of Mucin-1. Median CA 15–3 and CA 27.29 concentrations were significantly higher in patients with malignant disease (p< 0.001) than in patients with benign disease.

Paraffin-embedded tissue of 154 patients with ovarian carcinoma was available to determine Mucin-1 expression. The majority of patients presented with advanced stage disease at primary diagnosis. Median follow-up time was 11.39 years. Immunohistochemistry results for VU4H5 showed significant differences with respect to tumour grade, FIGO stage and overall survival. Patients with negative expression had a mean overall survival of 9.33 years compared to 6.27 years for patients with positive Mucin-1 expression.

**Conclusions:**

This study found significantly elevated Mucin-1 serum concentrations in ovarian carcinoma patients as compared to those women suffering from benign ovarian diseases. However, it needs to be noted that Mucin-1 concentrations in carcinoma patients showed a rather high variability. Results from immunohistochemistry indicate that Mucin-1 has a prognostic relevance in ovarian carcinomas when evaluating the expression by VU4H5 antibody.

## Background

Ovarian cancer is one of the most lethal malignancies. Patients with early stage ovarian cancer are often asymptomatic or report nonspecific symptoms so ovarian cancer is mostly diagnosed at an advanced stage [1,2].

Primary treatment includes operative cytoreduction and subsequent combined platinum-based chemotherapy. Though reported primary response rates are around 80%, ovarian cancer is the most lethal gynecological malignancy since 60-70% of the patients relapse or die within 5 years after primary diagnosis [1,3,4]. The prognosis of the disease could be improved by early detection, but this is difficult to achieve.

Mucin-1 (MUC1) is a heterodimeric protein complex that is normally located at the apical border of secretory epithelial cells. The N-terminal subunit is the mucin component of the protein consisting of variable numbers of tandem repeats that are linked with glycans. It is connected to the cell surface by association with the transmembrane C-terminal subunit. The physiological function of the protein is to build a barrier against toxins, microorganisms and other forms of stress [5]. During cell transformation and loss of polarity the protein expression is up-regulated. MUC1 is known to be over-expressed by various human carcinomas and is shed into the circulation where different epitopes can be detected in the serum of patients by specific anti-MUC1 antibodies [4,6,7]. CA 15–3 and CA 27.29 are available tumour marker assays for detecting MUC1. Monoclonal antibodies which are specific for the different tandem repeat units in the protein core of the MUC1 antigen are used in these kits and automated analysers produce results that are reliable [8]. Both markers are structurally similar and CA 15–3 is routinely utilised as a diagnostic and prognostic marker in breast cancer [9,10]. Clinical correlation studies comparing CA 15–3 levels and CA 27.29 levels in breast cancer patients typically show high correlation coefficients, suggesting that CA 27.29 would be suitable for routine use [11,12]. Recently published data confirmed this assumption [13], but the diagnostic relevance for patients with ovarian tumours of uncertain dignity remains unclear.

The Expression of MUC1 by immunohistochemistry (IHC) can also be detected by monoclonal antibodies. A large panel of epitopes exists to evaluate the prognostic value of MUC1 expression. VU4H5 and VU3C6 are both anti-MUC1 antibodies of the same isotype (mouse IgG1) and are directed at the core protein of MUC1. The anti-body VU4H5 was generated with a synthetic MUC1 peptide consisting of three tandem repeats as immunogen. Both antibodies, VU3C6 and VU4H5, were evaluated during the ISOBM TD-4 International Workshop on Monoclonal Antibodies against MUC1. They were confirmed in their MUC1 specificity. A major difference between the two antibodies is their epitope sequence. For VU3C6 the epitope sequence is **GVTSAPDTRPAP** and for VU4H5 it is **APDTRPAP**[14].

Overexpression of MUC1 has been reported in ovarian cancer, but the information is limited due to small numbers and the correlation between overexpression and prognosis remains unclear [15-17].

One aim of this study was to compare the concentrations of MUC1 in a cohort of patients with either benign or malignant ovarian tumours detected by CA 15–3 and CA 27.29. Another aim was to evaluate the MUC1 expression by IHC in a different cohort of ovarian carcinoma patients with respect to grade, stage and survival.

## Methods

### Patients

Patients from our study whose sera were tested for CA 15–3 and CA 27.29 underwent surgery at the Department of Obstetrics and Gynecology, Campus Innenstadt, LMU Munich between 2002 and 2006. Blood samples were obtained prior to surgery and were assigned to either the group of patients with benign (n=74) or malignant (n=44) disease of the ovary after histopathological examination. Histological evaluation and staging of tumour tissue was performed by an experienced gynaecological pathologist (D.M.) according to the criteria of the International Federation of Gynaecologists and Obstetricians (FIGO) and the World Health Organization (WHO).

Patients whose tissue was examined by IHC for MUC1 expression retrospectively had undergone surgery for primary ovarian carcinoma at the Department of Obstetrics and Gynecology, Campus Innenstadt, LMU Munich between 1990 and 2002. Patients with ovarian borderline tumours were excluded from the study. Again, histological evaluation and staging was performed by an experienced gynaecological pathologist. Clinical data was abstracted from patient charts and the tumour registry database. MUC1 expression was evaluated in terms of a possible correlation with tumour stage, grade and survival. The extent of the primary tumour (pT) is defined according to the UICC: pT1= the tumour is limited to the ovaries, pT2= the tumour has spread to the pelvis, pT3= the tumour has spread beyond the pelvis and/or to regional lymphnodes.

### Sample description

Tumour samples of 154 primary ovarian carcinoma patients were evaluated by IHC for MUC1. Median age at primary diagnosis was 58.8 years (range 18–88). The majority of patients presented with advanced stage disease at time of primary diagnosis [FIGO I: n=34 (22.1%), FIGO II: n=10 (6.5%), FIGO III: n=102 (66.2%), FIGO IV: n=3 (1.9%), missing: n=5 (3.2%)]. See Table 1 for detailed patient characteristics. Median follow-up time was 11.39 years. 26 patients relapsed and 91 died.

**Table 1:** Patient characteristics of ovarian carcinoma patients whose tissue samples were stained by immunohistochemistry for MUC1 expression

		
***Ovarian carcinoma patients (n)***		*154*
***Age at primary diagnosis (a)***		*58.8 (range 18–88)*
***Histology (%)***	*serous*	*70.8*
	*mucinous*	*8.4*
	*endometrioid*	*13.6*
	*clear cell*	*7.1*
***Tumor grade (%)***	*low grade*	*24.7*
	*intermediate*	*33.1*
	*high grade*	*34.4*
	*missing*	*7.8*
***Tumor stage (FIGO) (%)***	*I*	*22.1*
	*II*	*6.5*
	*III*	*66.2*
	*IV*	*1.9*
	*missing*	*3.2*

### Ethics approval

The study was approved by the local ethics committee of the Ludwig-Maximilians University Munich and was carried out in compliance with the guidelines of the Helsinki Declaration of 1975 (approval with the reference number 138/03). The study participants gave their written consent and samples and clinical information were used anonymously.

### Enzyme-linked-immunosorbent-assay (ELISA)

As previously described [13,18].

### Immunohistochemistry

IHC for MUC1 was performed as described elsewhere [19]. Antibodies used for staining were the anti-VU4H5 (mouse IgG; Zymed, Berlin, Germany) and anti-VU3C6 (1 mg/ml, mouse IgG; Serotec, Munich, Germany).

### VU4H5

In short, paraffin-fixed tissue sections were dewaxed with xylol for 15 minutes and placed into 100% ethanol. Blocking of the endogenous peroxidase was done by a combination of hydrogen peroxide and methanol for 20 minutes. Next, slides were dehydrated in descending concentrations of ethanol and then exposed for epitope retrieval for 10 minutes in a pressure cooker using sodium citrate buffer (pH 6.0) containing 0.1 M citric acid and 0.1 M sodium citrate in distilled water. After cooling, slides were washed twice in PBS. Non-specific binding of the primary antibodies was blocked by incubating the sections with "diluted normal serum" (10 ml PBS containing 150 μl horse serum; Vector Laboratories, CA) for 20 minutes at room temperature. Slides were then incubated with the primary antibodies at room temperature for 60 minutes. After washing with PBS, slides were incubated with the secondary antibody for 30 minutes and afterwards washed with PBS twice followed by incubation with ABC-complex for another 30 minutes. Visualization was conducted using substrate and chromagen 3,3'-diaminobenzidine (DAB; Dako, Glostrup, Denmark) for 8–10 min. Slides were then counterstained with Mayer's acidic hematoxylin and dehydrated in ascending concentrations of ethanol (50–98%). After xylol treatment, slides were covered.

MaCa 2402/02 served as a positive control for the MUC1 staining. For negative controls, the primary antibody was replaced with normal control serum IgG. Positive staining resulted in a brownish color, negative controls and unstained cells displayed a blue color.

### VU3C6

Paraffin-fixed tissue sections were dewaxed with xylol for 20 minutes and placed into 100% ethanol. Blocking of the endogenous peroxidase was done by a combination of hydrogen peroxide and methanol for 20 minutes. Next, slides were dehydrated in descending concentrations of ethanol and washed twice in PBS. Non-specific binding of the primary antibodies was blocked by incubating the sections with "diluted normal serum" (10 ml PBS containing 150 μl horse serum; Vector Laboratories, CA) for 20 minutes at room temperature. The remaining steps were the same as described for VU4H5.

**Figure 1:**
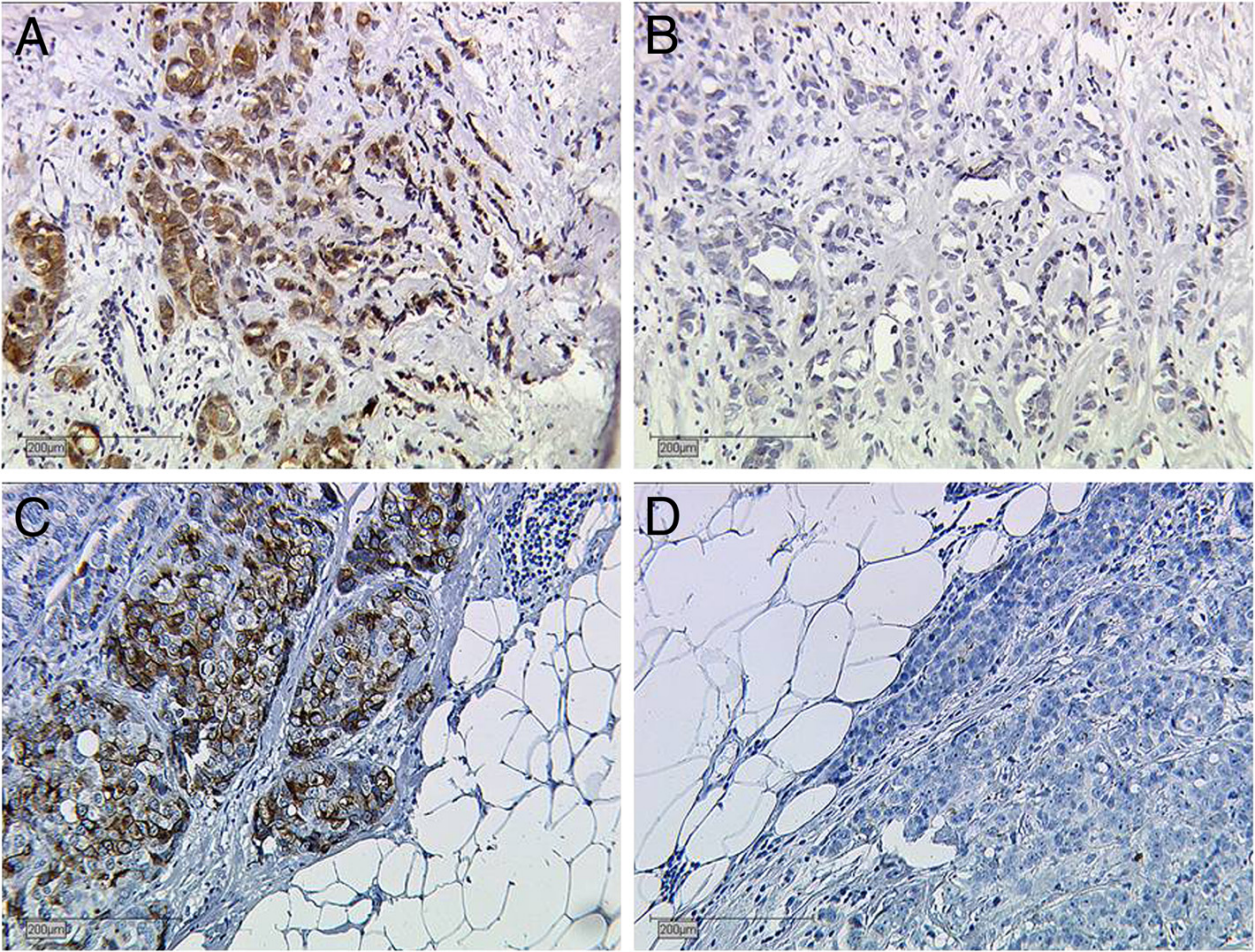
**Controls for VU4H5 and VU3C6. A**, posive and **B**, negative control for VU4H5. **C**, positive and **D**, negative control for VU3C6. Breast cancer tissue.

See Figure 1 for staining results of controls for each antibody.

### Immunohistochemical analysis

Slides were evaluated and digitalized with a Zeiss photomicroscope (Axiophot, Axiocam, Zeiss, Jena, Germany). Immunohistochemical staining was assessed using a semiquantitative score according to Remmele and Steger [20], comprising optical staining intensity (graded as 0 = no, 1 = weak, 2 = moderate, and 3 = strong staining) and the percentage of positively stained cells (0 = no, 1 = <10%, 2 = 10–50%, 3 = 51–80% and 4 = >80% cells). The values for staining intensity and the percentage of positively stained cells are multiplied, so a maximum score of 12 can be reached. According to Remmele and Steger, a score equal or less than 3 represents week staining and a score above 3 moderate or strong staining. We defined cases with an IRS of equal or less than 3 as negative and cases with an IRS of 4 or higher as positive which is consistent with previously published studies [21]. Slides were reviewed by two independent observers, including a gynecological pathologist (D.M.). One slide per case was evaluated by a magnification of 250x. In 11 cases (=7.05%), the evaluation of the two observers differed. These cases were jointly re-evaluated by the observers. After re-evaluation both observers came to the same result. The concordance before the re-evaluation was 145 (92.95%).

### Statistical analysis

Statistical analysis was performed by using SPSS 18.0 (PASW Statistic, SPSS Inc., IBM, Chicago, IL). Correlation analysis of MUC1 expression was performed for the histological subtype, tumour stage, grade and clinical data with the non-parametric Kruskal-Wallis rank-sum test and the non-parametric Spearman correlation coefficient. Kaplan-Meier curves were drawn for the comparison of survival times. Differences between survival curves were calculated using the chi-square statistic of the log-rank test to test curves for significance. Significance was assumed at p <0.05.

## Results

### CA 15–3 and CA 27.29 serum concentrations

Patients with benign ovarian disease (n=74) were further classified into 32 patients with retention cysts (including follicular cysts, corpus luteum cysts, endometriosis cysts, serous cysts), 38 patients with benign tumours (serous and mucinous cystadenoma, serous and mucinous cystadenofibroma, Brenner’s tumour, teratoma and fibroma) and four patients whose benign disease was not specified.

Those patients with ovarian carcinoma (n=44) were divided into serous (n=28), endometroid (n=15) and mucinous (n=1) histology.

The median concentration of CA 15–3 was significantly higher in patients with malignant disease (46 U/ml; range: 8.37-2990 U/ml) than in patients with benign disease (21 U/ml; range: 5.38-67.2 U/ml)(p<0.001). Table 2 shows median, minimum and maximum concentrations measured for each histological subtype.

**Table 2:** Median and range for CA27.29 and CA 15–3 within different the histological subtypes

**CA27.29**	**Serous (n=28)**	**Mucinous (n=1)**	**Endometrioid (n=15)**
**median**	27.835	12.26	43.31
**min**	6.21	-	21.99
**max**	329.31	-	55.5
**CA15.3**	**serous**	**mucinous**	**endometrioid**
**median**	34.1	11.7	59
**min**	8.37	-	32.5
**max**	1240	-	65.3

Evaluation of CA 27.29 also showed a significant difference with median concentrations of 16 U/ml (range: 4.00-48.77 U/ml) in patients with benign disease and 37 U/ml (range: 6.21-511.48 U/ml) in patients with ovarian carcinoma (p<0.001).

### MUC1 expression in ovarian carcinoma tissue

Immunohistochemical analysis resulted in 37 positive of 152 evaluable cases for VU3C6 and 106 positive of 150 evaluable cases for VU4H5. Of the 37 samples positive for VU3C6, 31 were positive for VU4H5, 4 were negative and 2 samples were technically not evaluable for VU4H5. The majority of positive samples (34) were of serous histology. All cases of clear cell and mucinous histology were negative for VU3C6 and only two cases of endometroid histology were positive for VU3C6.

The distribution of positive cases for VU4H5 regarding histological subtype was as follows: serous 82/107, clear cell 7/10, endometroid 13/21 and mucinous 4/12.

**Figure 2:**
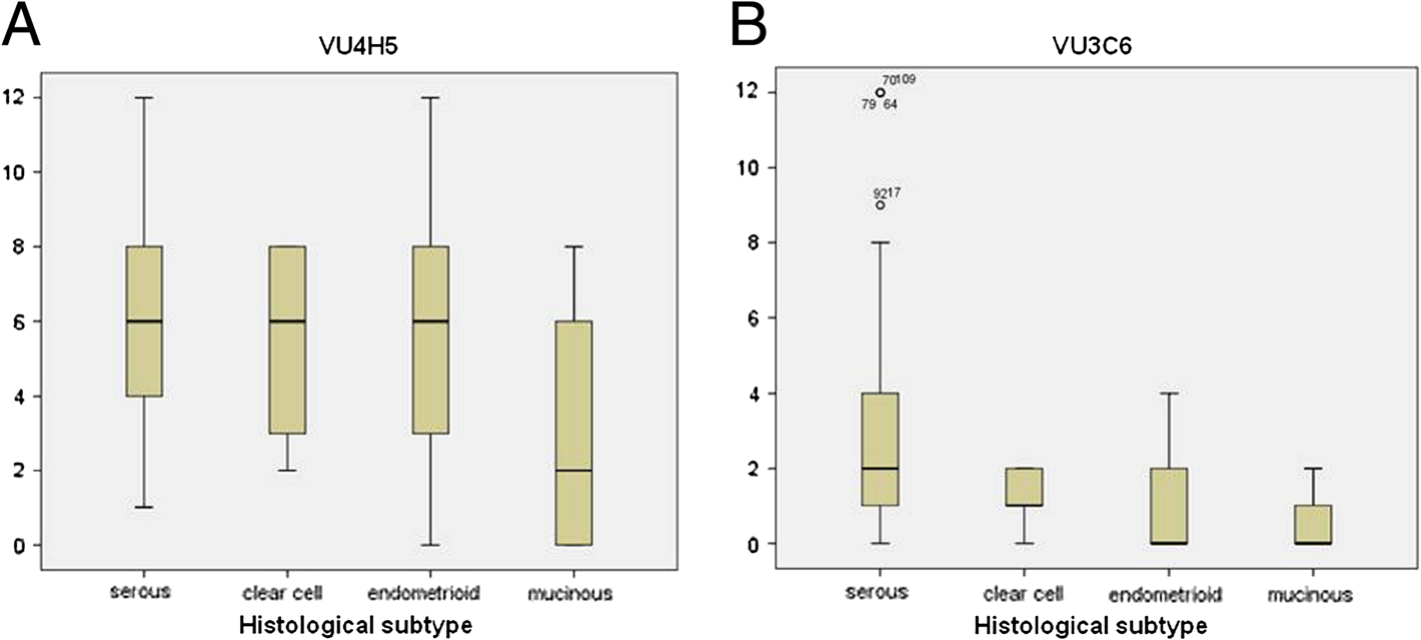
**MUC1 Expression within the different histological subtypes.** Significant differences of expression were found for VU4H5 (**A**, p=0.008) and VU3C6 (**B**, p<0.001). The boxes represent the range between the 25^th^ and 75^th^ percentiles with a horizontal line at the median. The bars delineate the 5^th^ and 95^th^ percentiles.

Median overall survival for all patients was 3.3 years (range 2.12-4.48). Figure 2 shows the expression of MUC1 in ovarian carcinoma subtypes in boxplots. There were significant differences in MUC1 expression between serous, clear cell, endometrioid or mucinous forms of ovarian carcinoma.

**Figure 3:**
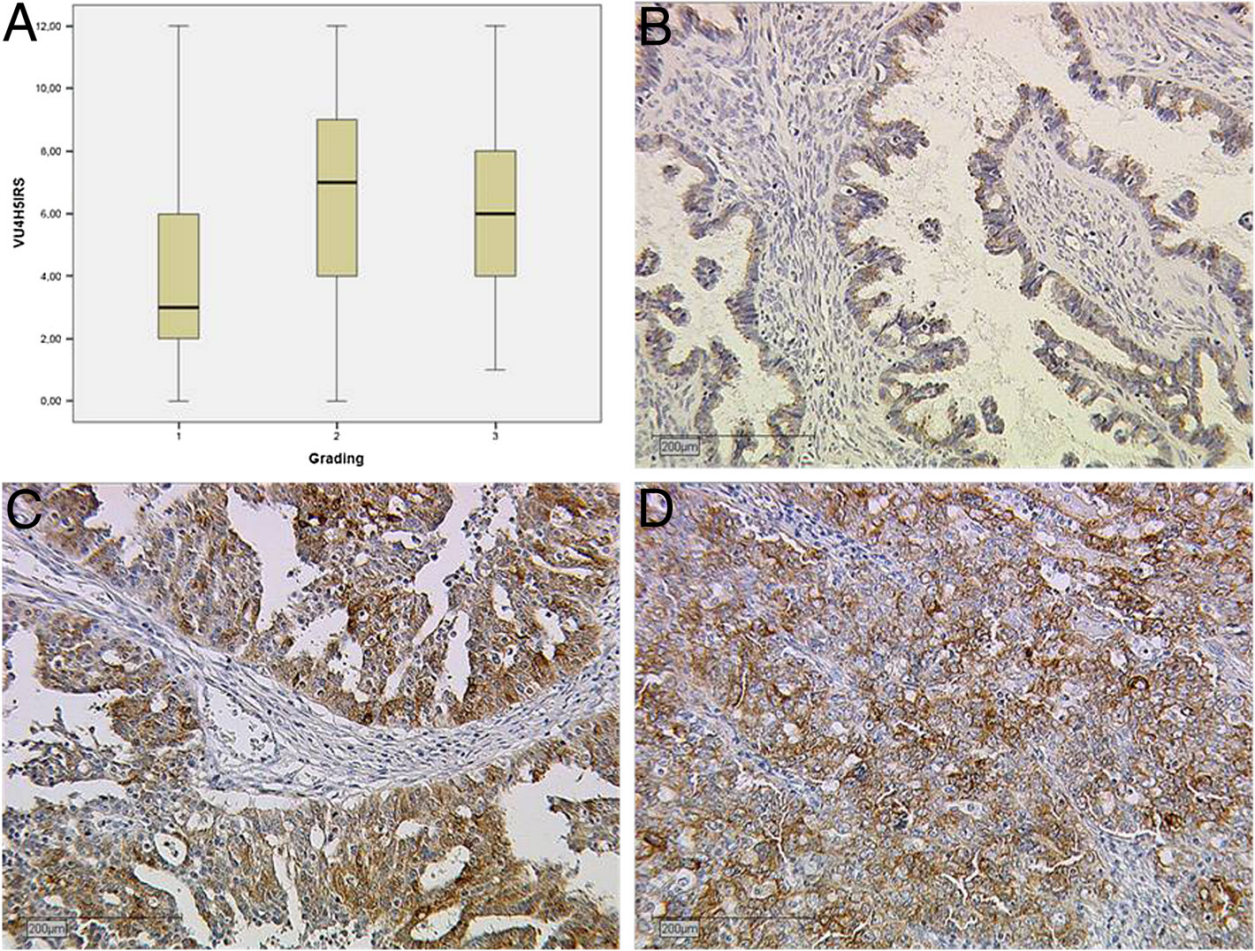
**Expression of MUC1 in ovarian carcinoma shown by grading.A**, Significant differences of expression were found for the VU4H5 epitope (p=0,003). **B**, week staining (IRS=2) for VU4H5 in a grade 1 carcinoma. **C** and **D**, strong staining (IRS=8) in cases with grading 2 and 3, respectively.

The correlation of the staining results for both antibodies with tumour grade, FIGO stage and pT-stage revealed results of varying significance: With respect to tumour grade we found a positive relationship between the tumour feature and MUC1 when samples were evaluated for VU4H5 (p=0.003), see Figure 3, but not for VU3C6 (p=0.104). The same positive relationship was found for VU4H5 regarding FIGO stage (p=0.047), but not for VU3C6 (p=0.115). A positive relationship for both antibodies was found when expression was correlated with pT stage (VU4H5: p=0.010; VU3C6: p=0.031), see Table 3.

**Table 3:** VU4H5 and VU3C6 were correlated to the extent of the primary tumour (pT), grade and FIGO stage; correlation is significant at the ** 0.01 level (2-tailed), * 0.05 level (2-tailed) and significant results are shown in bold

			**VU4H5**	**VU3C6**	**pT**	**grade**	**stage**
Spearman's rho	**VU4H5**	Correlation Coefficient	1.000	0.4860	0.209	0.24	0.162
		Sig. (2-tailed)		< 0.001**	0.010*	0.003*	0.047*
	**VU3C6**	Correlation Coefficient	0.486	1.000	0.174	0.131	0.127
		Sig. (2-tailed)	< 0.001**		0.031*	0.104	0.115

### Prognostic value of MUC1 expression

**Figure 4:**
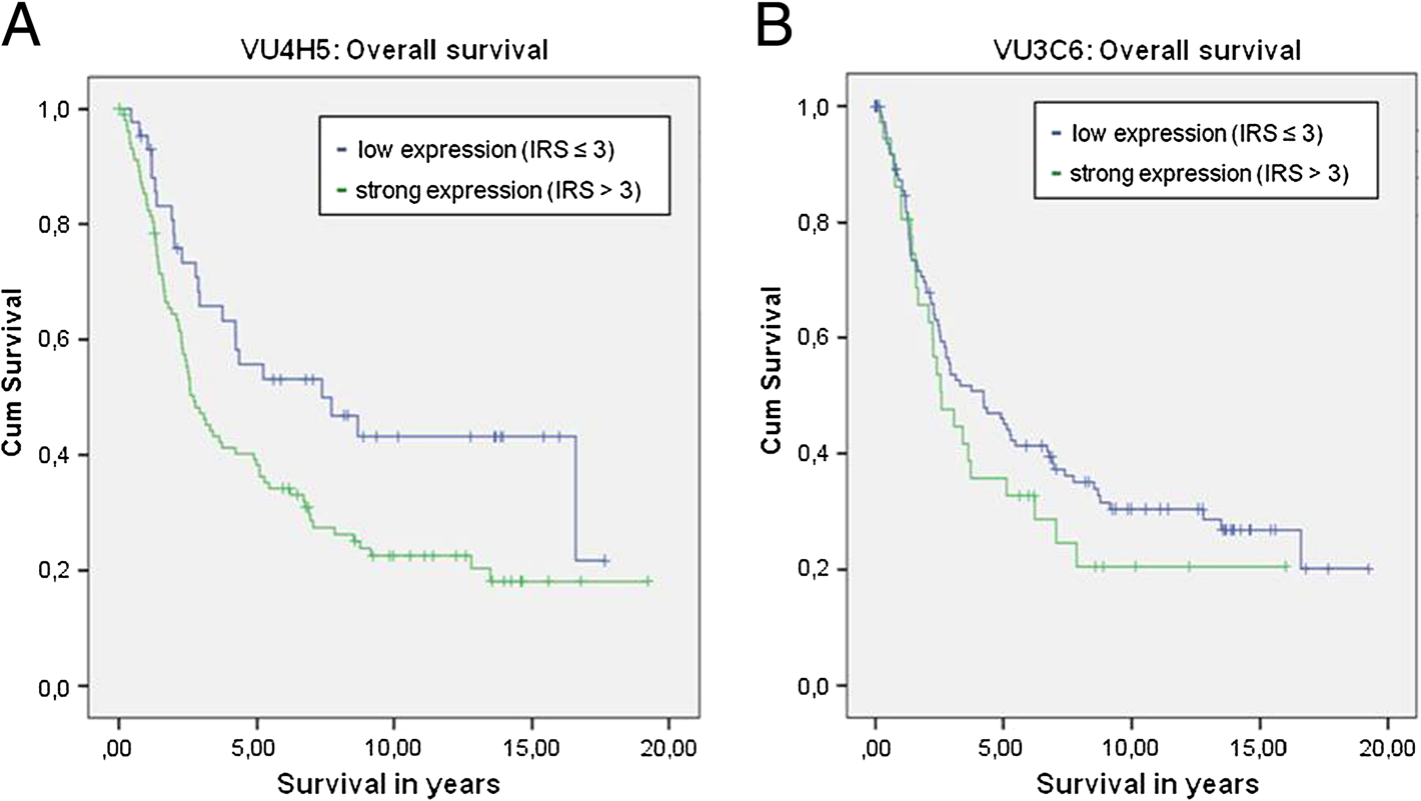
**Overall survival for all patients of our study cohort.** Kaplan-Meyer curves showing overall survival. Results for VU4H5 showed significant differences (p= 0.011, **A**), but VU3C6 did not (p=0.262, **B**).

**Figure 5:**
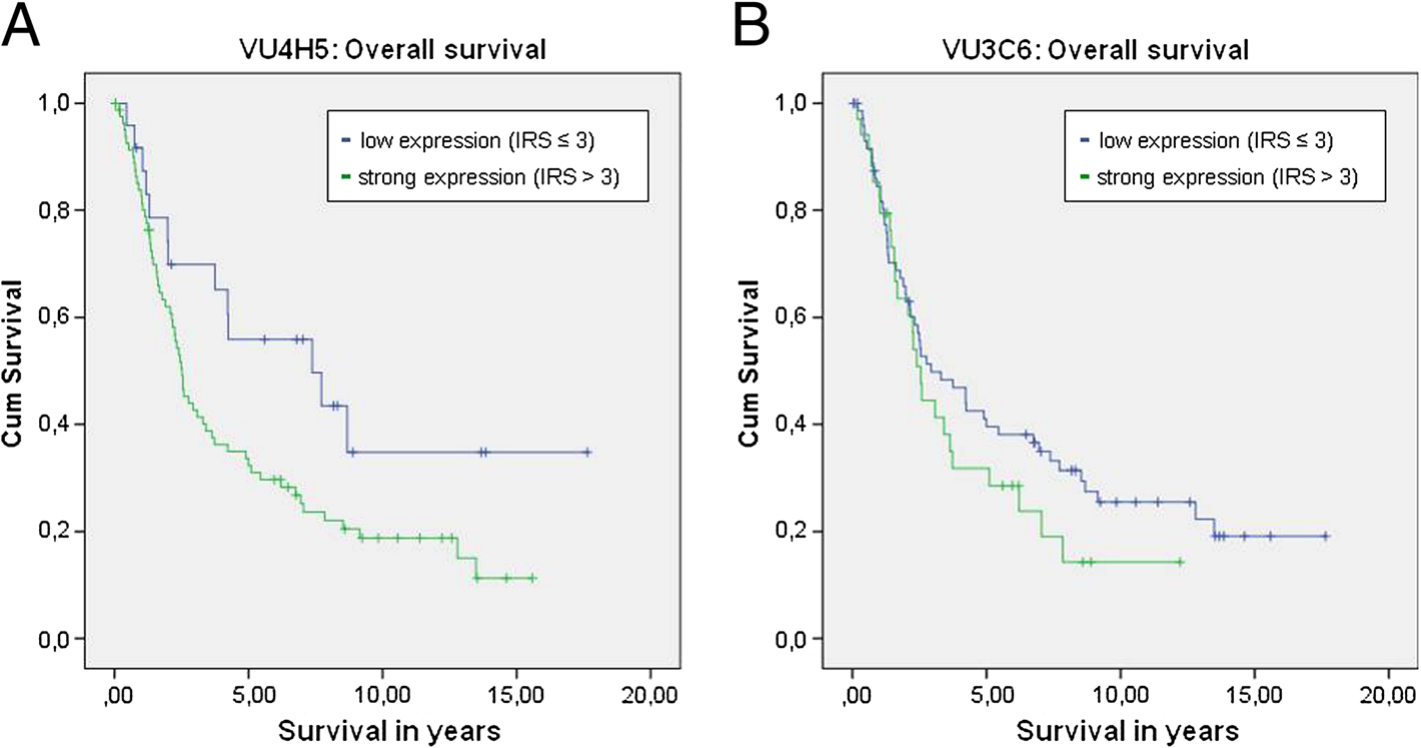
**Overall survival for the subgroup of serous ovarian carcinoma patients.** Kaplan-Meyer curves showing overall survival. Results for VU4H5 showed significant differences (p= 0.032, **A**), but VU3C6 did not (p=0.257, **B**).

Overall survival was correlated with the expression of VU3C6 and VU4H5. VU4H5 turned out to be a negative prognosticator in ovarian carcinoma patients. Patients with a negative VU4H5 expression showed significantly better mean overall survival (9.33 years; range: 7.09-11.57 years) when compared to patients with positive expression (6.27 years; range: 4.90-7.64 years), p=0.011. This applied to the serous subtype in particular. Mean overall survival for patients with serous MUC1 positive ovarian carcinoma was 4.98 years (range: 3.82-6.13) compared to 8.77 years (range: 5.69-11.85) for patients with negative expression, evaluated by VU4H5 (p=0.032). However in multivariate Cox-Regression analysis VU4H5 did not prove to be an independent prognostic marker in ovarian carcinoma cases. The expression of VU3C6 was not related to patients’ outcome, neither in the whole cohort (p=0.262) nor in the serous subgroup (p=0.257). See Figure 4 for survival curves of all patients and Figure 5 for survival curves of the subgroup of serous ovarian carcinoma patients.

## Discussion

The first part of this study evaluated serum concentrations of CA 15–3 and CA 27.29 measured in sera of patients with either benign or malignant tumours of the ovary. One aim of this study was to compare CA 15–3 and CA 27.29 in benign and malignant ovarian disease. Median concentrations showed significant differences between benign and malignant disease, but with high variability of the absolute value, so differentiation of benign and malignant disease by CA 15–3 or CA 27.29 does not seem possible. According to our results neither CA 15–3 nor CA 27.29 will have the potential to serve as a reliable routine tumour marker in ovarian cancer. Besides, the sample is not large enough to evaluate these markers for different histological subtypes of ovarian carcinomas since the number of cases of each histological subtype is very small.

In order to evaluate the potential of CA 15–3 to aid early detection of ovarian cancer, Shutter *et al.* investigated the combination of CA 15–3, CA 125, and CA 72–4 [22], but CA 15–3 was not able to improve the significance of this test. Other studies showed that MUC1 measured in sera of patients with platinum resistant disease inversely correlates with overall survival and might thus be useful as a prognostic marker [23,24]. However, MUC1 might be able to add diagnostic significance in addition to CA 125 testing which needs to be investigated in future studies.

The second part of this study evaluated MUC1 expression by IHC where two epitopes were targeted. VU4H5 is one of the most commonly used antibodies when targeting MUC1 and previous studies have shown a positive correlation for lymph node involvement and a higher staining intensity for higher grade breast cancer lesions [25]. Studies that evaluated the prognostic role of MUC1 in ovarian cancer also found a significant association with clinical-pathological features such as tumour stage, grade, residual disease status and presence of ascites [26]. Only the aberrantly glycosylated MUC1 is found to be over-expressed in ovarian cancer, whereas normal ovarian surface epithelium and serous cystadenomas do not express these epitopes [27]. Our results underline the possible prognostic potential of MUC1 in regard to tumour grade, FIGO stage and survival. Interestingly, this is only true when targeting the VU4H5 epitope as VU3C6 did not show significant differences for the mentioned variables.

As discussed above, MUC1 is a valuable tumour marker in breast cancer and early studies suggest it may be a useful target for vaccine strategies [20]. MUC1 as a target for immunotherapy has, however, encountered challenges. It is expressed on normal cells and so far we do not have the ability to distinguish between tumour-associated MUC1 and normal MUC1; the shed N-terminal subunit acting as a large pool to absorb the antibody [28]. However, in vitro studies on ovarian cancer cell lines were able to show increased sensitivity to docetaxel when combined with the monoclonal antibody MAb C595 and in vivo studies using a MUC1/docetaxel conjugate showed higher cytotoxicity than docetaxel alone in multidrug resistant ovarian cancer [29,30]. Another study compared patients that were treated with a Yttrium-labeled monoclonal antibody recognising an extracellular portion of MUC1 versus controls treated by standard therapy alone. In this study no significant difference in terms of time to relapse and overall survival was found [31]. Our study shows a worse outcome for patients with high expression of MUC1 in ovarian carcinoma and thus supports its potential for targeted therapy. Future clinical studies will have to find out the most efficient conjugate.

## Conclusions

In this study, the median expression of MUC1 was significantly different in the serum of patients with benign and malignant ovarian disease, but the variability of the absolute value in patient’s sera is high so that a clear differentiation between malignant and benign disease is not possible. Our results from IHC indicate a prognostic relevance of MUC1 in ovarian carcinoma when evaluated by the VU4H5 antibody. New therapeutic strategies may also directly target MUC1 and increase efficacy and specificity of anticancer treatment. However, our study has some limitations since we investigated only two out of a variety of existing anti-MUC1-antibodies. Ovarian cancer is a heterogeneous disease. Our study cohort consists of different numbers of serous, endometroid, clear cell and mucinous ovarian carcinoma cases. Future studies need to investigate other existing antibodies in regard to their specificity and sensitivity of detecting MUC1 epitopes and should focus on differences regarding each tumour type.

## Abbreviations

MUC1: Mucin-1; IHC: Immunohistochemistry; UICC: Union for International Cancer Control.

## Competing interests

The authors declare that they have no competing interests.

## Authors’ contributions

VE has made substantial contributions to analysis and interpretation of data and drafted the manuscript. ALS carried out the immunoassay. SH and ML participated in the design of the study and helped with the statistical analysis. HE as a physician with scientific expertise and native speaker improved the wording and helped to revise the manuscript. UA, MGB, BR, and MK have made substantial contributions to acquisition of data and helped to draft the manuscript. CK carried out immunohistochemistry. DM and UJ participated in the study design and coordination and helped to draft the manuscript. All authors read and approved the manuscript.

## Pre-publication history

The pre-publication history for this paper can be accessed here:

http://www.biomedcentral.com/1471-2407/12/600/prepub
